# Nanometer Gap in Electromechanical Converters—A Way to Achieve an Extremely High Energy Density

**DOI:** 10.3390/mi10110746

**Published:** 2019-10-31

**Authors:** Igor L. Baginsky, Edward G. Kostsov

**Affiliations:** Institute of Automation and Electrometry, Siberian Branch, Russian Academy of Sciences, 630090 Novosibirsk, Russia; baginsky@iae.nsk.su

**Keywords:** energy converter, electrostatics, nanogap, ferroelectric, limiting energy density

## Abstract

The analysis of electromechanical energy converters based on metal-thin film ferroelectric (with a large specific capacitance)-nanogap-moving electrode structures was performed. It was shown that the density of the energy being converted and its absolute value increase with the decreasing gap value between the surfaces of the ferroelectric and the metallic moving electrode up to nanometer values. The effects limiting the growth of this energy were established, and the limiting value of the energy density transformed in the nanogap of these structures was determined to be about 1.6 × 10^10^ J/m^3^, which is 4 orders of magnitude higher than the energy density in inductive converters. The experimental verification of this model based on the data for micromotors fabricated on these structures is given.

## 1. Introduction

Electromechanical energy converters (motors and generators) that are widely used in all fields of human activity and in industry are divided into three classes: inductive, piezoelectric, electrostatic (electrical) machines, in which direct and reverse conversion of magnetic or electric field energy to mechanical energy is performed. The specific and absolute power of energy converters are determined by the energy density of the field in the working gap.

In the best inductive converters the magnetic field energy density in the gap reaches *W*_V_ = 1/2 *B*^2^/*μ*_0_ = 4 × 10^5^–10^6^ J/m^3^ (where *μ*_0_ is the magnetic constant) at the maximum possible values of magnetic induction, *B*, (of the order of 1–1.5 T) for magnetic circuits created on the basis of ferromagnets. In this case minimum gaps between the rotor and stator, determined by the production technology, are of the order of fractions of a millimeter, less than 100–300 μm [1].

Piezoelectric energy converters with respect to field energy density are comparable to electromagnetic ones, see, for example, devices based on PZT ceramics [2]. In such converters, the working gap is the material of the piezoelectric itself. The energy density in it can be high due to the large value of its dielectric constant, *ε*, as well as large values of the electromechanical coupling coefficient, of the order of 0.5–0.8. However, for such converters, the value of the maximum energy density being converted is not limited by the maximum field that can be formed in the dielectric without breakdown, but it is limited by the value of the mechanical stress of the material, above which an irreversible deformation of the piezoelectric occurs: *σ* = *F*_e_/*S* (where *F*_e_ is the elastic force, *S* is the converter area): *W*_V_ = 1/2 *σ*^2^/*E*_Y_ (where *E*_Y_ is the Young’s modulus of the material) [3].

In capacitive electrostatic energy converters, the specific volume energy density, *W*_V_, is also determined by the electric field strength in the working gap *E*_e_:

(1) $$W_{V}=\frac{\varepsilon_{0}E_{e}{}^{2}}{2}=\frac{\varepsilon_{0}V_{e}{}^{2}}{2d_{e}{}^{2}}=\frac{C_{e}V_{e}{}^{2}}{2d_{e}}$$

where *V*_e_ is the voltage applied to the gap, *d*_e_ is the gap width, *C*_e_ = *ε*_0_/*d*_e_ is the specific (per unit area) gap capacity, *ε*_0_ is the dielectric constant of vacuum. The total energy per unit area of the structure *W*_S_ is equal to:

(2) $$W_{S}=\frac{C_{e}V_{e}{}^{2}}{2}=\frac{\varepsilon_{0}V_{e}{}^{2}}{2d_{e}}$$

Classical electrostatic, capacitive, energy converters have not yet found wide application because of the low specific energy density *W*_V_ ≈ 40 J/m^3^. *W*_V_ is determined by the low electric field strength *E*_e_ in the working gap of the metal-gap-metal structure (MGM), which is limited by the value of the breakdown field strength of the gap *E*_b_. This value is known to be equal to 3 × 10^6^ V/m for air at atmospheric pressure.

An increase in the field strength in the gap can be achieved by introducing an additional dielectric layer into the MGM structure, i.e., by the use of metal-dielectric-gap-metal structures (MDGM). The introduction of a dielectric layer, which suppresses the development of breakdown, makes it possible to increase the voltage applied to the structure to several hundred volts with small gaps, of the order of 10–30 μm, see, for example, [4]. Due to this, an energy density comparable to one characteristic for large inductive converters was reached.

The work of capacitive electrostatic energy converters is based on the shift of the moving electrode (ME) in the metal- dielectric- gap- ME structure either under the action of electric field forces (motor [5,6]) or under the action of mechanical force against the electric field forces (generator [7]). The shift of the moving electrode can be carried out both in in-plane structures (in this case, the electrode overlap area is modulated without changing the gap between the ME and the dielectric), and in out-of-plane ones (the gap changes at the constant electrode area). It should be noted that in known MDGM structures further growth of the density of converted energy due to decrease of the gap width up to the nanometer scale is not realized, because in these conditions the field in the gap does not practically increase. Under small values of gap width comparable or less than dielectric film thickness the voltage *V* applied to the structure is distributed in such a way that only a small part of it is applied to the gap. Therefore, the only way to increase the energy density in the gap of MDGM structures is to increase the applied voltage *V* to the values, limited by the breakdown of the dielectric layer, see, for example, [8,9].

Obviously, it is necessary to use dielectric materials with a high value of ε and high breakdown voltage values in order to decrease the voltage drop in the dielectric when the width of the gap is decreased to nanometer scale. Such properties are characteristic for ferroelectric thin films, for which the breakdown field strength reaches 100–350 V/μm with *ε* being in the range of 1000–5000, see, for example, the review in [10]. High values of *ε*, more than 2000, are also characteristic for barium-strontium niobate (BSN) films with the composition Ba_0.5_Sr_0.5_Nb_2_O_6_ [11], which were deposited by us earlier by high-frequency sputtering on the surfaces of silicon and sapphire substrates with a sublayer of a conducting electrode indium tin oxide (ITO) or Pt, they have breakdown voltages of about 100 V/μm.

The purpose of this work is to determine the limiting density of electromechanical energy conversion in metal-ferroelectric-gap-metal structures (MFGM), with the smallest possible gap (nanometer-wide) between the metal and the ferroelectric.

## 2. Materials and Methods

The analysis of possible limiting operating modes of electromechanical capacitive energy converters based on MDGM-structures in order to determine the maximum energy density in the gap, and, accordingly, the maximum value of the converted energy, will be carried out using well-known literature data describing both the breakdown of nano-gaps and the current flow through them.

An experimental verification of the obtained results will be carried out on the basis of our previously obtained results on prototyping and investigation of film petal micromotors made on the basis of the metal-thin film NBS-gap-free thin metal film (petal) structures.

## 3. Results

### 3.1. Estimation of the Limiting Energy Density in Electromechanical Converters

The maximum energy density *W*_S,max_ in the capacitive energy converter under consideration is determined by the following parameters: the minimum gap between the surfaces of the moving electrode and the ferroelectric *d*_e,min_ (and, accordingly, the maximum capacitance of the gap layer *C*_e,max_) and the maximum voltage *V*_max_. *V*_max_ is the voltage that can be applied to a MFGM-structure without its breakdown and without the onset of currents in the gap, that result in charge accumulation on the surface of the ferroelectric and, consequently, in the field decrease in the gap *E*_e_. 

The breakdown of both air and vacuum gaps in the metal-gap-metal (MGM) structure was studied quite intensively. А large number of papers devoted to the breakdown of micron and submicron gaps was published earlier, see, for example, reviews in [12,13,14].

**Figure 1:**
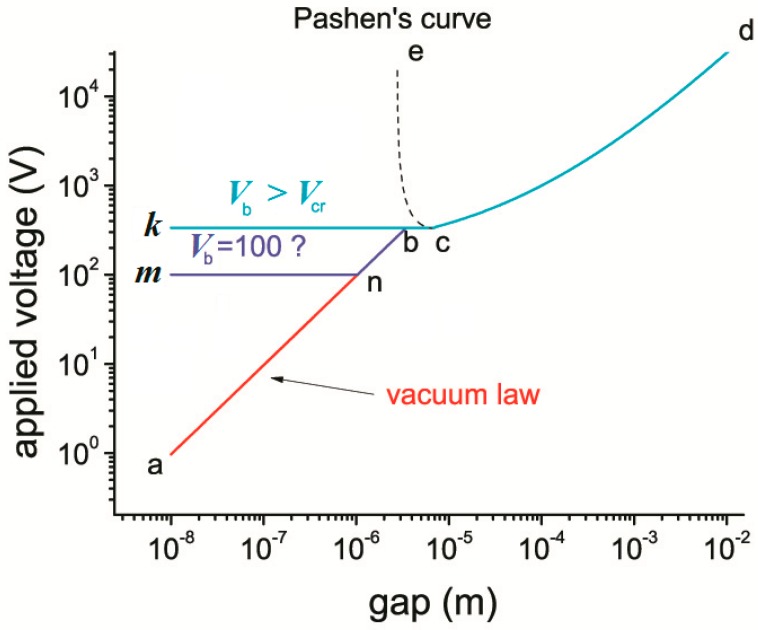
The dependence of the critical voltage on the size of the gap d_e_ at atmospheric air pressure: in metal-gap-metal structure (MGM) - (curve abcd) and metal-ferroelectric-gap-metal structures (MFGM) structures (mnbcd and kbcd curves). ecd is Paschen curve.

Studies of the MGM structures show that at atmospheric pressure, starting from gaps of 7 μm and less, there is a deviation from the classical Paschen breakdown law (ecd curve, Figure 1): The dependence of the critical voltage *V*_cr_, which in this case is equal to the breakdown voltage of the structure *V*_b_, from the gap width *d*_e_ is a plateau (bc curve on the *V*_cr_(*d*_e_) curve). Then At *d*_e_ < 3 μm *V*_cr_ decreases linearly with decreasing *d*_e_ from *V*_i_ = 330 V in the plateau region to 1 V at *d*_e_ = 10 nm, curve ab [12] (see curve abcd in Figure 1). This behavior of *V*_cr_ is explained by the change in the breakdown mechanism: from impact ionization described by the Paschen law (curve ecd) to the breakdown by the vacuum mechanism (curve ab), which is typical for small gaps at which the ionization length exceeds the width of the gap. Under the conditions of the vacuum mechanism the breakdown is determined by a sharp increase in the concentration of electrons injected from micro-spikes on the electrode surface [15]. In particular, when micro-spikes are smoothed out by melting, the breakdown voltage increases several times [16,17], remaining relatively small in magnitude.

For the out-of-plane structures nanometer-sized minimum gaps are possible. Let us consider the features of the operation of such energy converters.

**Figure 2:**
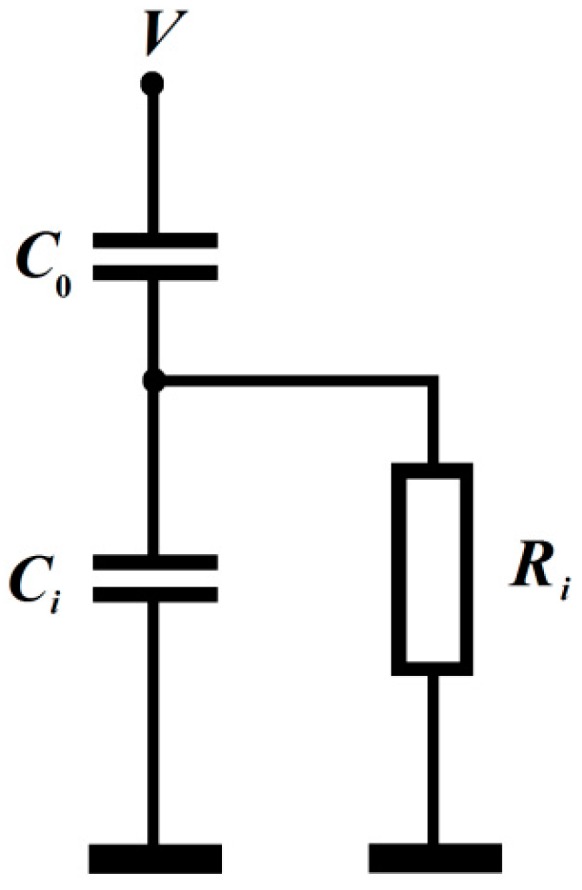
Equivalent circuit of the MFGM structure. *R*_i_ - leakage resistance of the insulator (ferroelectric).

MDGM-structure can be represented as a serial connection of two capacitances of the dielectric layer *C*_F_ and the air gap *C*_e_, Figure 2. In this case, the voltage drop across the air gap is expressed as:

(3) $$V_{e}=V\frac{C_{F}}{C_{e}+C_{F}}=\frac{V}{\frac{d_{F}}{\varepsilon d_{e}}+1}=V\frac{d_{e}}{d_{e}^{*}}$$

(4) $$d_{e}^{*}=d_{F}/\varepsilon +d_{e}$$

In the case when *C*_F_<<*C*_e_ or *d*_F_/*ε*>>*d*_e_, we have *V*_e_<<*V*. So, the most part of the voltage applied to the structure falls on the dielectric layer. Since the dielectric constant of linear dielectrics usually does not exceed 2–10, if the dielectric thickness is about 2–20 μm, which is necessary to prevent breakdown of the structure, reducing the gap to values less than 1 μm will reduce the fraction of voltage applied to the gap. In this case, as *d*_e_ decreases, the energy density *W*_V_, calculated in accordance with Equation (1), does not increase, since the field in the gap does not change and is equal to:

(5) $$E_{e1}=\frac{V}{d_{F}/\varepsilon }$$

The total energy per unit area *W*_S_ (see Equation (2)) decreases in accordance with the decrease in the voltage drop across the gap:

(6) $$V_{e1}=V\frac{\varepsilon d_{e}}{d_{F}}$$

Thus, to increase the energy density, as well as the total energy of a capacitive energy converter by reducing the gap between the surfaces of the moving electrode and the dielectric the opposite condition should be fulfilled: *C*_F_ >> *C*_e_ or *d*_F_/*ε* << *d*_e_. In this case, almost all the voltage is applied to the gap: *V*_e_ ≈ *V*. Since the thickness of the dielectric should be in the range of a few microns (according to the conditions for maintaining the electrical strength of the structure), its dielectric constant must be higher than 1000. This means that in order to reach high energy in converters based on MDGM structures, it is necessary to use ferroelectric thin films with a high dielectric constant value as a dielectric layer. In this case the *d*_e_ value can be reduced to 5–20 nm.

**Figure 3:**
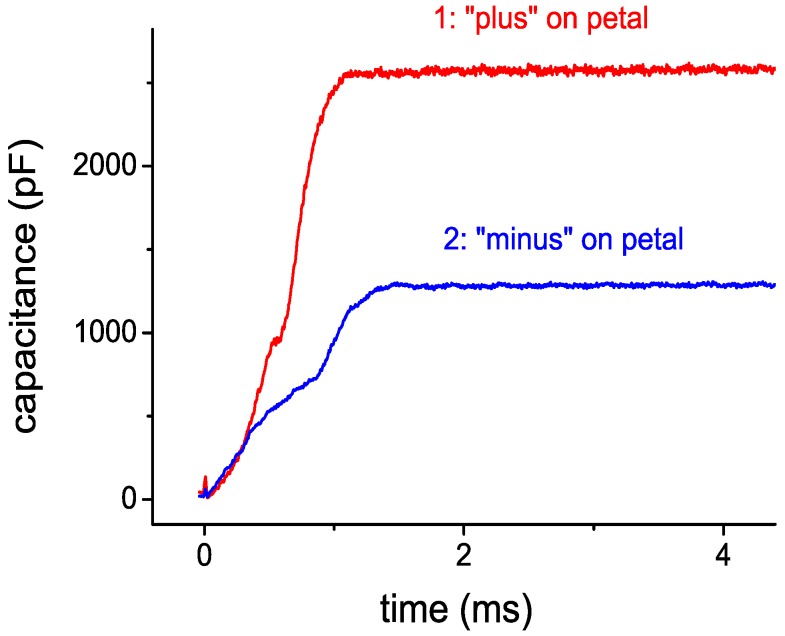
Polarity effect in the process of moving electrode (ME) attraction to the surface of a ferroelectric film in the MFGM structure at application of voltage pulse with an amplitude of 50 V. 1: positive bias on ME, 2: negative.

Let us estimate the maximum electric field strength *E*_max_ in the nano-gaps of these structures and the maximum energy density. It is clear that this value should be dependent on the polarity of the voltage applied to the moving electrode if the values of field strength in the nanogap are very high. At negative voltages the current of autoelectron emission flowing from the ME surface through a tunnel-transparent gap to the surface states of the ferroelectric film may occur, giving rise to field screening in the nano-gap by charge accumulation on this surface. Such a process was previously observed experimentally in the study of the memory effect in structures based on thin films of silicon nitride with a sublayer of a tunnel-transparent layer of silicon dioxide [18]. When electrostatic micromotors have been investigated (see [19]), this effect manifested itself in charge accumulation at the gap-ferroelectric interface even when sufficiently small (less than 50 V) negative voltages were applied to the moving electrode of the MFGM structure. In particular, the force of electrostatic attraction of ME to the surface of a ferroelectric decreases leading to a corresponding decrease in the capacitance of the structure, as compared with the case of positive displacement, see Figure 3. Note that for micromotors the effect of charge accumulation in the ferroelectric layer causes the slider to slow down up to total shutdown.

If the mobile electrode is at a positive potential relative to the opposite electrode, then the mechanisms limiting the magnitude of the field in the gap are the effects of field evaporation of metal atoms from the electrode surface and electron tunneling directly from the ferroelectric valence band [20,21]. According to estimates, the magnitude of the field, starting from which these effects are significant, is *E*_cr_ = 6 × 10^10^ V/m [20,21].

It is easy to show that for parameters characteristic for a ferroelectric film: *ε*/*d* = 10^9^ (μm)^−1^ or *C*_F_ = 8.85 × 10^−3^ F/m^2^, the field in the gap of 5 nm in width can reach 10^10^–6 × 10^10^ V/m. Then, in the absence of field screening in the gap (for example, when the structure is placed in vacuum) and with a sufficiently high breakdown voltage of the ferroelectric layer, the maximum density of electrical energy in the nanogap of the MFGM structure is equal to *W*_V,max_ = 1.6 × 10^10^ J/m^3^, see Equation (1), and the energy per unit area can reach *W*_S,max_ = 80 J/m^2^, respectively.

Thus, the value of *W*_V,max_ for the considered capacitive converters exceeds the energy density of the inductive converters by 4 orders of magnitude. It is also easy to show (see Equation (2)) that for conventional electrostatic generators on MGM structures the value of total energy *W*_S,max_, comparable to that estimated above for MFGM-structures, can be reached only with an interelectrode gap equal to 2 m and the applied voltage of 10^6^ V.

### 3.2. Peculiarities of Operation of the Electromechanical Transducer in the Modes of Limiting High Energy Density in the Nanogap

Let us consider electromechanical transducers in which the gap is modulated by the movement of ME from the minimum (nanometer) value *d*_e,min_ to a certain value *d*_e,max_. Depending on the conditions in the environment, there are two possible modes of operation of the energy converter based on the MFGM structure, which we will further call as “atmospheric” and “vacuum”. For the atmospheric mode, when the minimum gap changes from nanometer values of *d*_e,min_ during ME oscillations with amplitudes *d*_e,max_ exceeding *d*_e,cr_ = 3 μm, the air ionization in the gap occurs, starting from *V*_e_ = *V*_i_, see Figure 1, which is absent in the vacuum mode. The vacuum mode can also manifest itself at atmospheric air pressure, when the impact ionization length exceeds the maximum width of the gap, i.e. with the ME oscillation amplitude being less than *d*_e,cr_.

Under conditions of the atmospheric mode, and when the breakdown voltage of a ferroelectric *V*_b_ is less than the minimum voltage on the Paschen curve *V*_i_, the dependence of the maximum possible voltage in the MFGM structure on the gap size is described by the curve mnbcd, Figure 1. If *V*_b_ > *V*_i_, then as the gap increases starting from 3 μm to 7 μm air is ionized in the gap by the impact ionization mechanism. It leads to the formation of a screening charge on the surface of the ferroelectric, which reduces the power consumption of the converter. In this case, the electric field in the gap, see [22], is expressed as:

(7) $$E_{e}=Q_{S}/\varepsilon_{0}=-C(V-V_{P})/\varepsilon_{0}$$

where *Q*_S_ is the charge on ME, *V*_P_ = *Q*_P_/*C*_F_, *Q*_P_ is the charge accumulated at the gap-dielectric boundary. The attraction force of the electric field acting on the moving electrode is:

(8) $$F=\frac{1}{2}Q_{S}E_{e}=\frac{Q_{S}{}^{2}S}{2\varepsilon_{0}}$$

and the energy converted in the structure is:

(9) $$W_{S}=\frac{Q_{S}{}^{2}}{2C}=\frac{C{\left(V-V_{P}\right)}^{2}}{2}$$

It is clear, that the process of charge accumulation *Q*_P_ will be stopped, when the voltage drop across the gap *E*_e_ × *d*_e_ (see Equation (7)) reaches the impact ionization potential *V*_i_, when:

(10) $$V=V_{\mathrm{cr}}=\frac{d_{e}{}^{*}}{d_{e}}V_{i}$$

Then:

(11) $$E_{e}{}_{,\mathrm{cr}}=-\frac{d_{e}{}^{*}}{d_{e}}CV_{i}/\varepsilon_{0}$$

Therefore, in the case under consideration, the energy conversion is described by the curve kcd, Figure 1, and the maximum energy produced during the conversion cycle is:

(12) $$W_{S,\max}=\frac{C_{\max}V_{i}{}^{2}}{2}{\left(\frac{d_{e}{}^{*}}{d_{e}}\right)}^{2}$$

where:

(13) $$C_{\max}=\frac{C_{e,\max}C_{F}}{C_{e,\max}+C_{F}}=\frac{\varepsilon_{0}S}{d_{e}{}^{*}}$$

Thus, the critical voltage *V*_cr_ is equal to *V*_b_ when ferroelectric films with a high value of *ε* are used in MFGM structures and under the conditions *V*_b_ < *V*_i_,. As the applied voltage reaches this value, a breakdown of the structure occurs. When *V*_b_ > *V*_i_, the critical voltage is calculated by Equation (10), it is equal to the ionization potential *V*_i_. When this voltage is exceeded, impact air ionization in the gap begins, leading to the formation of a charge that screens the field in the gap.

Let us estimate the maximum density of electrical energy that can be reached in converters based on MFGM structures. Assuming that *d*_e_ = 5 nm, *d* = 1 μm, *ε* = 1000 (or *ε*/*d* = 10^9^ m^−1^, *C*_F_ = 8.85·10^−3^ F/m^2^) and taking into account that *V*_i_ = 330 V, we obtain: *d*_e_^*^ = 6 nm, *C* = 1.48 × 10^−3^ F/m^2^ and *W*_S,max_ = 80 J/m^2^ and *W*_V,max_ = 1.6 × 10^10^ J/m^3^, correspondingly.

Note that these estimates of the limiting energies *W*_S,max_ and *W*_V,max_ coincide with their values for the vacuum breakdown mode and at a gap size of 5 nm, when the energy is limited by some critical field in the gap *E*_cr_. When *E*_e_ > *E*_cr_ the evaporation of metal atoms from the surface of PE by the electric field starts. According to [20,21], the value of this field can be estimated as 6 × 10^10^ V/m. In particular, for atoms of copper, lanthanum and zinc, it is equal to 3 × 10^10^ V/m, for tungsten atoms it reaches 10^11^ V/m. It can be noted that there are no restrictions on the value of *W*_S,max_ in this mode associated with the ionization of air in the gap, so it increases with the growth of the gap width, see Equation (2):

(14) $$W_{S,\max}=\frac{\varepsilon_{0}E_{e,\mathrm{cr}}{}^{2}d_{e}}{2}$$

with the bulk energy density *W*_V,max_ = 1.6 × 10^10^ J/m^3^ being constant.

In this case, the parameters limiting the growth of *W*_S,max_ are both the electrical strength of the ferroelectric, limiting the voltage applied to the MFGM structure, and the increase in the conduction current in the gap, which screens the field in it. The conduction current through the nanogap increases starting from some critical width *d*_e,cr_, at which the ion-electron emission current arises [23]. The mechanism of this emission lies in the injection of electrons from the valence band of a ferroelectric into the vacuum gap when a metal ion pulled by the field from the moving electrode strikes the surface of the ferroelectric. Since it is known [23] that such emission begins at an ion velocity *v* of the order of 10^7^ m/s, then, based on the energy balance, the critical gap value will be equal to:

(15) $$d_{e,\mathrm{cr}}=\frac{mv^{2}}{2eE_{\mathrm{cr}}}$$

In particular, for the copper atom *d*_e,cr_ = 50 nm.

Thus, in the vacuum mode, it is possible to increase, practically by an order of magnitude, the values of the energy *W*_S,max_, compared to the atmospheric mode.

### 3.3. Examples of Practical Implementation of Energy Converters on MFGM-Structures. Experimental Evaluation of Maximum Energy Density

**Figure 4:**
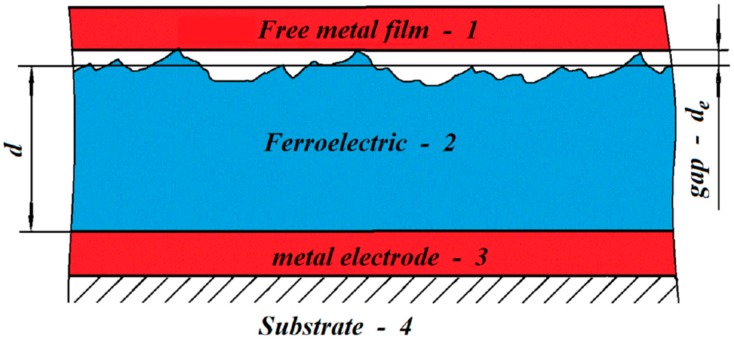
Schematic of the structure metal (1) - gap (thickness *d*_e_) - dielectric (BSN) (2) thickness *d*_F_ - metal (3), (4) - substrate - silicon.

**Figure 5:**
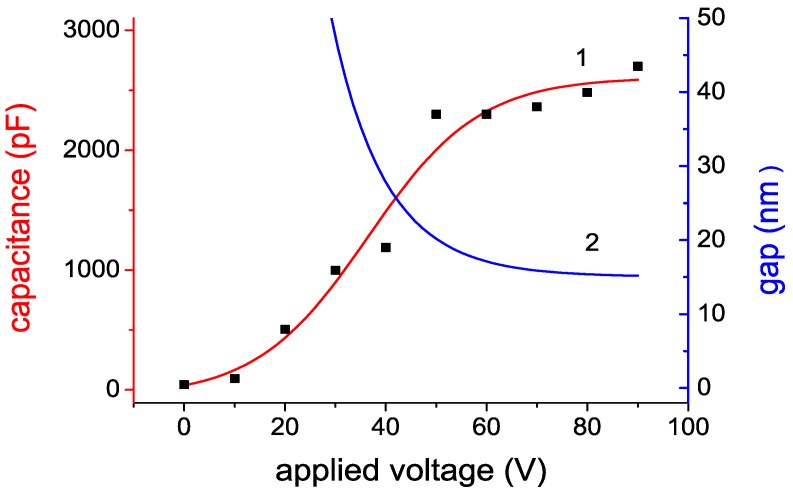
The change in the capacity of the MFGM structure and the size of the gap depending on the applied voltage (*d*_F_ = 2.4 μm, *ε* = 3500).

An example of a MFGM structure with a nano-gap created using the electrostatic attraction of a free metal film to the surface of a ferroelectric [19,24] is shown in Figure 4. The minimum size of the gap is determined both by the dimensions of microspikes on the surface of the ferroelectric (ranging from 5 to 50 nm), and by the force of electrostatic attraction of the metal film to the surface, which depends on the amplitude of the applied voltage, Figure 5, see [25].

**Figure 6:**
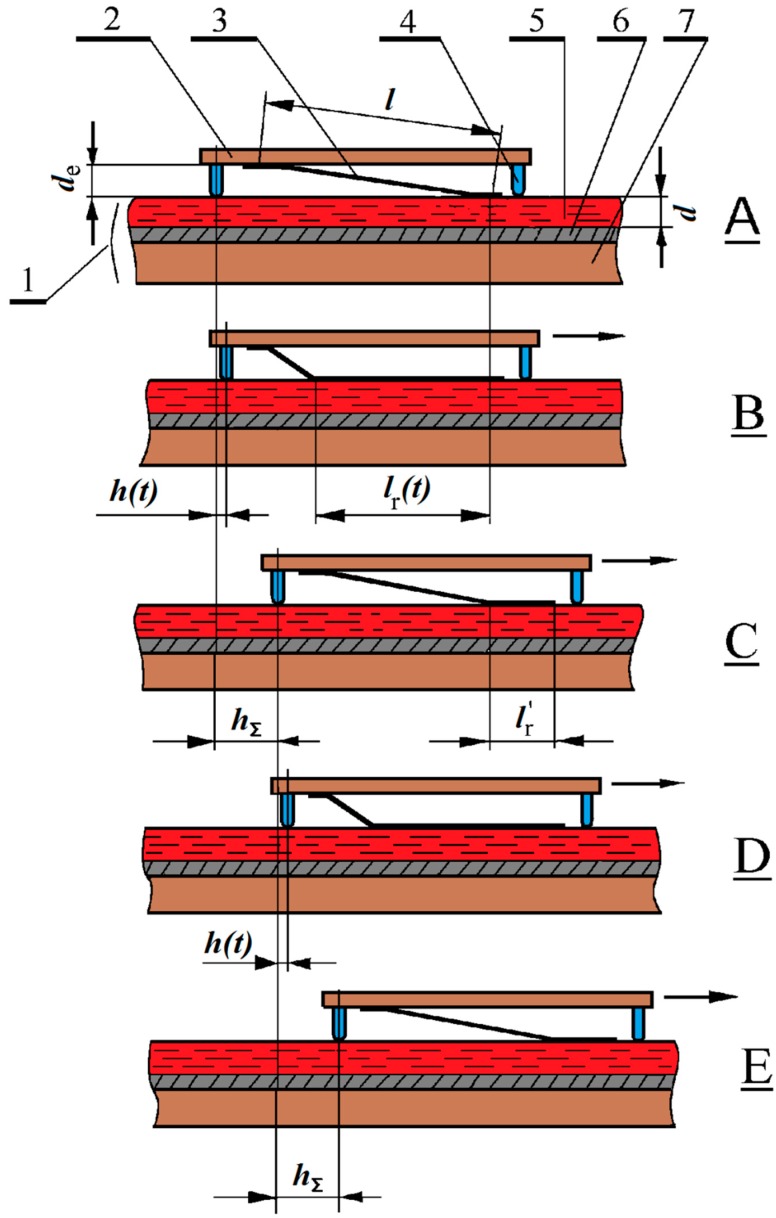
A scheme illustrating for the motion effects for the petal micromotor. 1 – stator, consisted of ferroelectric film (5), deposited on silicon substrate (7) with metallic electrode sublayer (6), 2 – moving substrate (glass), 3 – petal (copper berillium), 4 – guides (schematically shown). **A** – initial state and position, *t* = 0; **B** – the state and position at the end of the first voltage pulse, *t* = *t*_p_, the moving substrate (slider) is shifted to *h*(*t*) and the petal is rolled to the length *l*_r_(*t*); **C** – the state and position, corresponding to the time *t* = *T* (*T* is the period of voltage pulses). The slider is shifted to the distance *h*_Σ_ by inertia, a part of the petal *l*_r_^’^ is still attached to the ferroelectric surface; **D** – the state and position at the end of the second pulse, analogous to the view **A**, total shift is *h* + *h*_Σ_; **E** – the state and position, corresponding to the time *t* = 2*T*, analogous to the view **C**, toal shift is 2*h*_Σ_.

The design of an electrostatic micromotor, which was developed by us earlier [19,24,25,26,27]. Some of these designs were patented [28,29]. Figure 6, can serve as an example of an energy converter based on a MFGM structure with a nano-gap that develops high mechanical forces.

The operation of such micromotors is based on the successive attraction by electrostatic forces (section by section) of a free metal film (petal) to the ferroelectric surface with an electrode underlayer deposited on the substrate resulting in the formation of a nanometer gap, see Figure 6.

The stationary plate (stator) I consists of the silicon substrate 7 with the electrode 6 and ferroelectric film ***5*** deposited on it’s surface. The moving plate (rotor) 2 is separated from the stator by the gap 4. The metallic petals 3 with the length equal to I are synthesized on the rotor’s surface. Rotor moves with respect to the stator along the guides 4.

When the pulse is applied between the petal 3 having it’s initial position **A** and the electrode 6, the part of the petal is rolled to the ferroelectric surface (state B). The metallic film bends, stretches and transfers the movement to the plate 2, thus carrying out the electromechanical energy conversion. The rolling length *l*_r_*(t)* increases as the voltage pulse acts, and, therefore, rotor’s shift *h(t)* varies with the voltage pulse duration *t*. After the end of voltage pulse action the petal under the effect of the elastic force comes to it’s initial position A (with a single voltage pulse) or to the new position C, typical to the continuous rotor movement (when a series of the pulses is applied to the sample),and the rotor is moving by means of inertia to the distance *h*_Σ_. The duration of this process defines space between the voltage pulses, i.e. the maximum frequency of these pulses, of order (*ρ* is the specific weight of petal material, *d*_P_ is te petal width, *l* is it length, *E*_Y_ – Yong’s modulus), and, consequently, the power output of the micromotor. When the second voltage pulse is applied to the sample the moving plate with the petals makes one more step and comes to the position D. After the end of the pulse under the effect of the inertia the rotor comes to position E (and the petals’ configuration comes to the state analogous to C).

(16) $$f_{0}\approx 0.162\frac{d_{P}}{l^{2}}\sqrt{\frac{E_{Y}}{\rho }}$$

When the third and further pulses come, the movement occurs in the similar way: ftom position B to position C with the corresponding movement of the plate 2 This work describes the basic characteristics of the electrostatic micromotors and the peculiarities of their functioning in the step mode.

**Figure 7:**
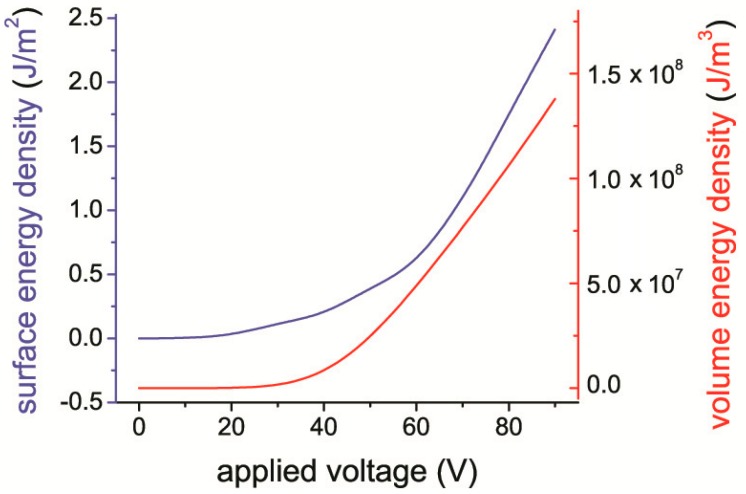
The energy density in the nanogap of the MFGM structure, depending on the applied voltage. (*d*_F_ = 2.4 μm, *ε* = 3500).

Based on the parameters indicated in Figure 5, it is possible to estimate the energy accumulated in the gap of the structure under consideration, for example, at a voltage of 90 V, see Figure 7. It is equal to 2.5 J/m^2^. Extrapolating the value of the total energy *W*_S_*(V)* to voltage equal to *V*_i_ = 330 V, and assuming that *W*_S_ increases quadratically with increasing voltage, we get *W*_S,max_ = 32 J/m^2^. Note that this is a lower estimate, since the dependence *W*_S_*(V)* is stronger than a quadratic one, because the gap decreases with increasing voltage.

We also have found experimentally that these micromotors reached an energy density of *W*_S_ = 1 J/m^2^ with a *V* value of 50 V [19]. Assuming that this energy density with the rise of voltage increases proportionally to *V*^2^ and extrapolating this value to *V*_max_ = 330 V, we get *W*_S,max_ = 40 J/m^2^, which is two times less than the value of *W*_S,max_ theoretically estimated above.

The value of the maximum power density, per unit weight of the structure, which can be reached for the described micromotors, is calculated as:

(17) $$P_{m}=\frac{W_{S,\max}f_{0}}{hk\rho_{1}}$$

where *f*_0_ is determined by Equation (17), *ρ_1_* is the specific weight of the material of the substrates of stator and slider, *h* is the total thickness of the substrates, *k* is the surface filling ratio by metal petals. Taking *k* = 0.8, *d*_P_ ≈ 1.5 μm, *l* = 100 μm, *h* = 1 mm and taking into account that the petals are made of beryllium bronze and the substrates are made of silicon, we get *f*_0_ ≈ 100 kHz and *P*_m_ = 4.3 × 10^6^ W/kg. This value of *P*_m_ exceeds the specific power of inductive engines by 3 orders of magnitude: the maximum value of *P*_m_ for high-speed inductive engines is 5 × 10^3^ W/kg, [30,31].

Estimates of the strength of the petals necessary to reach the limiting density of the converted energy *W*_S,max_ = 80 J/m^2^, show that their thickness can be within 1.5–2 microns. Such thicknesses can be easily achieved technologically.

## 4. Discussion

1. It has been shown that the creation of MFGM structures based on thin films of ferroelectrics with high electric strength and with a large dielectric constant of more than 1000 makes it possible to achieve high values of electric fields in nanogaps between the surfaces of a moving electrode and a ferroelectric, up to 6 × 10^10^ V/m. The energy density in the nanogap of electromechanical transducers based on such structures reaches 1.6 × 10^10^ J/m^3^, which exceeds this parameter in known inductive and piezoelectric transducers by more than 4 orders of magnitude.

2. It has been shown that during electromechanical energy conversion in these structures, the maximum value of energy, up to 80 J/m^2^, is achieved by forming a gap 5 nm in width and using a voltage of 330 V and a ferroelectric film thickness of 4 μm.

3. The effects limiting the maximum energy density in these structures are impact ionization of the air in the gap and the breakdown of the structure. It has been established that in the vacuum mode, the mechanism limiting the increase in energy is the field evaporation of metal atoms from the surface of the moving electrode.
